# Effect of COVID-19 vaccination on the timing and flow of menstrual periods in two cohorts

**DOI:** 10.3389/frph.2022.952976

**Published:** 2022-07-25

**Authors:** Alexandra Alvergne, Ee Von Woon, Victoria Male

**Affiliations:** ^1^School of Anthropology and Museum Ethnography, University of Oxford, Oxford, United Kingdom; ^2^Department of Metabolism, Digestion and Reproduction, Imperial College London, Chelsea and Westminster Hospital, London, United Kingdom

**Keywords:** SARS-CoV-2, COVID-19, vaccination, menstrual cycle, menstruation, withdrawal bleed

## Abstract

COVID-19 vaccination protects against the potentially serious consequences of SARS-CoV-2 infection, but some people have been hesitant to receive the vaccine because of reports that it could affect menstrual bleeding. To determine whether this occurs we prospectively recruited a cohort of 79 individuals, each of whom recorded details of at least three consecutive menstrual cycles, during which time they each received at least one dose of COVID-19 vaccine. In spontaneously cycling participants, COVID-19 vaccination was associated with a delay to the next period, but this change reversed in subsequent unvaccinated cycles. No delay was detected in those taking hormonal contraception. To explore hypotheses about the mechanism by which these menstrual changes occur, we retrospectively recruited a larger cohort, of 1,273 people who had kept a record of their menstrual cycle and vaccination dates. In this cohort, we found a trend toward use of combined hormonal contraception being protective against reporting a delayed period, suggesting that menstrual changes following vaccination may be mediated by perturbations to ovarian hormones. However, we were unable to detect a clear association between the timing of vaccination within the menstrual cycle and reports of menstrual changes. Our findings suggest that COVID-19 vaccination can lengthen the menstrual cycle and that this effect may be mediated by ovarian hormones. Importantly, we find that the menstrual cycle returns to its pre-vaccination length in unvaccinated cycles.

## Introduction

As the UK COVID-19 vaccination program was rolled out to younger participants, the UK medical regulator's Yellow Card surveillance scheme, to which healthcare professionals and members of the public can report suspected vaccine side effects, increasingly received reports from people who had noticed a change to their menstrual cycle following vaccination. By May 18, 2022, 39,839 individuals had made such reports to the Yellow Card surveillance scheme (1). It is important to note that most people who report such a change following vaccination find that their period rapidly returns to normal (2) and extensive investigation has found no evidence that COVID-19 vaccination adversely impacts female fertility (3–11). Nonetheless, people are concerned by these reports. Investigating the potential link between COVID-19 vaccination and menstrual changes is important for maintaining public trust in the vaccination program and, if a link is found, to allow people to plan for potential changes to their cycles (12).

Until recently, very little was known about how vaccination could affect the menstrual cycle, but there was some evidence that HPV vaccination may be associated with heavier or irregular periods (13). One study has recently been published specifically addressing the potential link between COVID-19 vaccination and changes to menstrual cycle length, finding the first dose of vaccine had no significant effect on timing of the subsequent period, while the second dose was associated with a delay of 0.45 days. Individuals who received both doses of the vaccine in a single cycle experienced a 2.32 day delay to their next period (14). In all groups, cycle lengths returned to normal by two cycles after vaccination. There is also evidence that viral infection, including with SARS-CoV-2 itself, can alter the menstrual cycle (15, 16). Taken together, these studies suggest that immune stimulation can affect the menstrual cycle. Biologically plausible mechanisms by which this could occur include effects mediated by immunological influences on the hormones driving the menstrual cycle (17, 18) or by immune cells in the lining of the uterus, which are involved in the cyclical build-up and breakdown of this tissue (19).

To address the potential link between COVID-19 vaccination and changes to menstrual bleeding, we recruited two cohorts. The first cohort consisted of 79 individuals recruited before receiving either their first or their second dose of the COVID-19 vaccine, who kept a daily record of their vaginal bleeding and any vaccine side effects they experienced. We used this prospective cohort to determine whether COVID-19 vaccination is associated with changes to menstrual timing or flow, as well as to determine whether menstrual effects are more likely to occur in those who experience other common side effects of vaccination. The second cohort consisted of 1,273 individuals who had already received their COVID-19 vaccination but had a record of the dates of their periods and the date or dates on which they received the vaccine. We used this retrospective cohort to explore specific hypotheses about mechanisms by which COVID-19 vaccination could cause changes to menstrual periods.

## Methods

### Ethical approval

Data collection for both cohorts was approved by the Research Governance and Integrity Team at Imperial College London, study number 21IC6988.

### Prospective cohort

**Figure 1:**
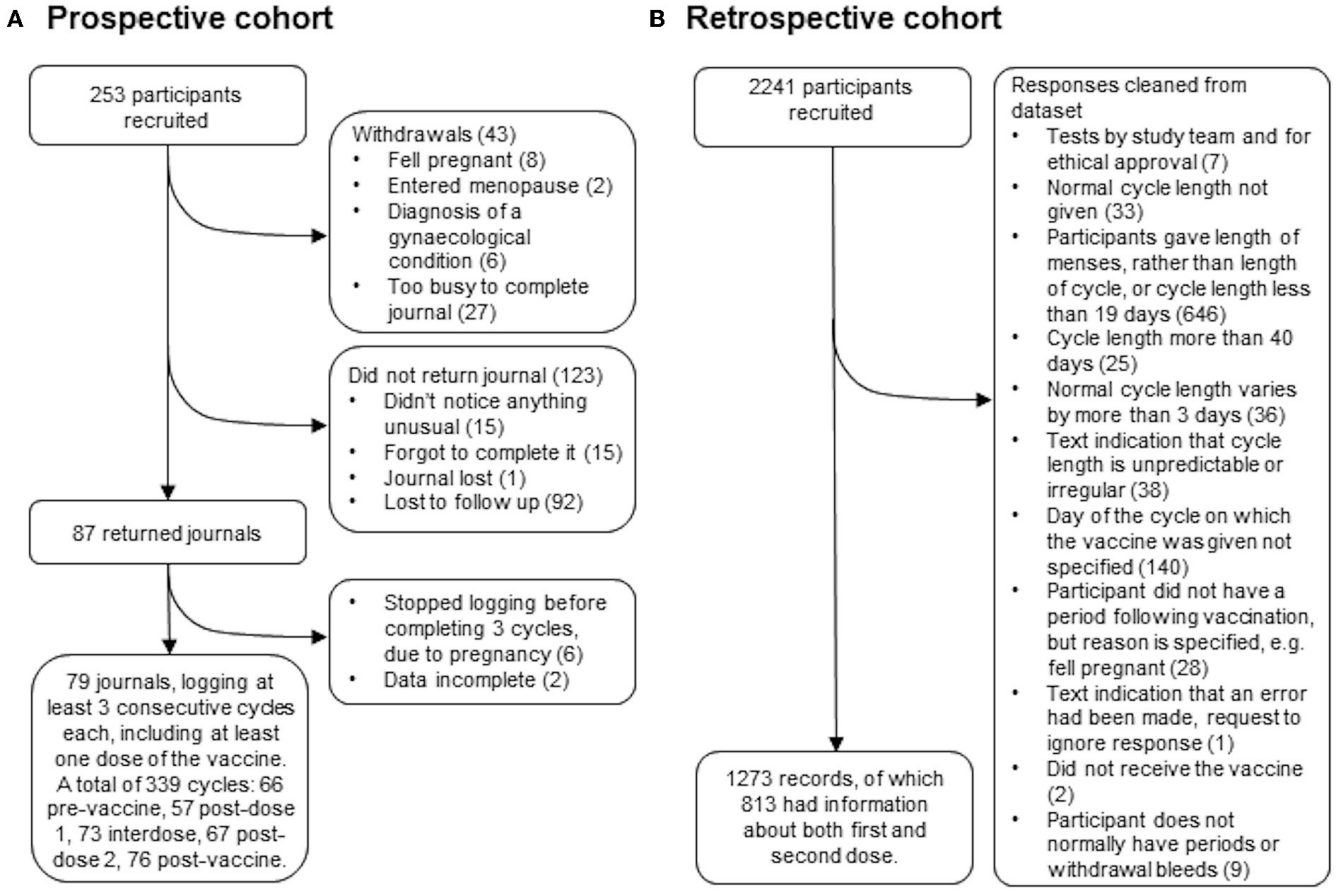
STROBE flowcharts for the prospective **(A)** and retrospective **(B)** cohorts.

Two hundred fifty-three people who were over 18, have periods or withdrawal bleeds and who were planning to receive either their first or their second dose of the COVID-19 vaccine were recruited by advertising on social media and in newsletters with a largely female readership in the UK. Of these, 43 withdrew for reasons including pregnancy, having entered the menopause, having received a diagnosis of a gynecological condition that they felt was affecting their periods, or not having the time to journal every day. Of the remaining participants, 87 returned their journals and 79 of these journals logged at least three consecutive cycles, including at least one in which a dose of the vaccine was given. Those who did not return their journals were contacted up to twice to find out why they had not completed their journals: 31 participants responded and 92 were lost to follow up (Figure 1A).

The data collection tool recorded the participant's age, reproductive history, use of hormonal contraception during the study period, breastfeeding during the study period and whether they have ever been diagnosed with a menstrual or gynecological condition (Table 1). Participants then completed a daily journal in which they reported their bleeding as “heavier than usual for this day of my cycle”, “normal”, “lighter than usual for this day of my cycle”, “spotting (normal for me)”, “spotting (not normal for me)” or “no bleeding”. For each cycle, participants also reported whether their period had come on the day they had expected it, and if not how many days early or late it was. Participants noted the day on which they received a dose of the vaccine, which brand they received, whether it was the first or second dose, and for seven days afterwards recorded whether they experienced any of the following: sore arm, fever, fatigue, headache, body aches. The data collection tool is available at https://osf.io/upbyg/.

**Table 1:** Demographic characteristics of participants in the prospective and retrospective cohorts.

	**Prospective**	**Retrospective**
Age (Median, IQR)	30 (27–35)	33 (29–29)
Cycle length, days (Median, IQR)	28 (27–30)	28 (27–30)
Hormonal contraception
No hormonal contraception	63 (79.7%)	1,117 (87.6%)
Combined pill	7 (8.9%)	53 (4.2%)
Progesterone only pill	3 (3.8%)	17 (1.3%)
IUS	5 (6.3%)	47 (3.7%)
Contraceptive patch	1 (1.3%)	3 (0.2%)
Contraceptive implant	0 (0%)	2 (0.2%)
Contraceptive injection	0 (0%)	2 (0.2%)
Vaginal ring	0 (0%)	2 (0.2%)
Other	0 (0%)	10 (0.8%)
Not specified	0 (0%)	5 (0.4%)
Previous diagnoses
Abnormal menstrual bleeding	4 (5.1%)	22 (1.7%)
Heavy menstrual bleeding	12 (15.2%)	150 (11.8%)
Endometriosis	3 (3.8%)	60 (4.7%)
Polycystic ovaries	7 (8.9%)	87 (6.8%)
Uterine fibroids	5 (6.3%)	21 (2.4%)
Currently breastfeeding
Yes	6 (7.6%)	89 (7.1%)
No	73 (92.4%)	1,179 (92.5%)
Not specified	0 (0%)	5 (0.4%)
Previous pregnancies
0	45 (57.0%)	572 (45%)
1	15 (19.0%)	219 (17.2%)
2	7 (8.9%)	220 (17.3%)
3 or more	8 (10.1%)	252 (19.8%)
Not specified	4 (5.1%)	10 (0.8%)
Vaccine
Pfizer	65 (82.3%)	778 (61%)
Moderna	11 (14.0%)	136 (10.7%)
AstraZeneca	3 (3.8%)	346 (27.1%)
Janssen	0 (0%)	8 (0.6%)
Not specified	0 (0%)	5 (0.4%)

The timing of each cycle was designated as “0” when the period or withdrawal bleed started on the expected day, with negative values indicating days early and positive values days late. For each cycle, a “flow score” was calculated by assigning “no bleeding” as 0, “spotting (normal for me)” and “spotting (not normal for me)” as 1, “lighter than usual” as 3, “normal” as 5 and “heavier than usual” as 7: responses were totaled for the first seven days of the cycle. “Spotting (normal for me)” and “spotting (not normal for me)” were assigned the same value for this analysis because the two indicate similar levels of flow. Where participants logged more than one cycle in a particular category (for example, pre-vaccine, interdose or post-vaccine cycles) the mean of the timing and flow scores was taken. For each dose of the vaccine, a “side effect score” was calculated by the total number of days that the participant responded “yes” to each of the side effect questions.

### Retrospective cohort

Two thousand two hundred forty-one people who were over 18, had received at least one dose of a COVID-19 vaccine, have periods or withdrawal bleeds and who have a record of the dates of these, and the date or dates on which they received the vaccine were recruited by advertising on social media and in newsletters with a largely female readership in the UK. Participants used a web-based form, which was open between July 27 and October 17, 2021, to anonymously report their age, length of their normal menstrual cycle, whether they use any hormonal contraception, whether they are breastfeeding, whether they have ever been diagnosed with a menstrual or gynecological condition and, for each dose of the vaccine, which brand they received, on which day of their cycle they were vaccinated and details of how the timing and flow of their next period compared to what they normally experience. The data collection tool is available at https://osf.io/6jf4u/. After data cleaning, 1,273 records remained, of which 813 had data for both the first and second dose of the vaccine (Figure 1B). Participant details are given in Table 1.

In order to increase the power to detect changes from the norm for each individual, participants who indicated that their cycle usually varied by more than 3 days were excluded: where participants gave their normal cycle length as a range (of 3 or fewer days), the median value was used for analyses that involved cycle length. For examination of the effect of the day of the cycle on which the vaccine was given, the day of ovulation was estimated by cycle length – 14, based on the observation that the luteal phase of the menstrual cycle is normally constant at around 14 days (20). The day on which the vaccine was given, relative to the predicted day of ovulation, was therefore calculated by cycle day of vaccination – (cycle length – 14).

### Statistical analysis

For the prospective cohort, changes in cycle timing and flow score were assessed using a mixed-effects model with the Geisser-Greenhouse correction for sphericity and Tukey's test for multiple comparisons: corrected *p*-values (*p*') are reported. Sensitivity analyses were carried out by assuming that every participant who had not returned their journal had experienced scores for every cycle with the same distribution as the pre-vaccine cycle scores recorded by those who did return their journals: the distribution was produced by randomly sampling scores from the pre-vaccine cycle data distribution for each missing value. Associations between side effect, timing and flow scores were assessed using Spearman's correlation with the Holm-Bonferroni sequential correction for multiple hypothesis testing: corrected *p-*values (*p*') are reported. These analyses were performed with Prism version 9.0.0.

For the retrospective cohort, independence between the following pairs of variables were determined using Chi squared tests: 1. Brand of vaccination and timing of next period; 2. Brand of vaccination and flow of next period. For tests 1 and 2, respondents who had either received Janssen or did not specify the brand of vaccine received (*n* = 13) were excluded from the analysis. 3. Use of hormonal contraception and timing of next period; 4. Use of hormonal contraception and flow of next period. For tests 3 and 4, respondents who did not clearly specify the form of contraception they use (*n* = 15) were excluded from the analysis. 5. Timing of vaccination and timing of next period; 6. Timing of vaccination and flow of next period. For tests 5 and 6, participants using hormonal contraception or who did not clearly specify the form of contraception they use, were excluded (*n* = 156); menstrual cycle days on which fewer than 5 respondents had been vaccinated were excluded (*n* = 16 respondents vaccinated more than 17 days before the predicted day of ovulation), as were days on which the respondent was already overdue for their period at time of vaccination (*n* = 99), since these respondents would, by definition, report that their period was later than usual. The Holm-Bonferroni sequential correction was used to correct for multiple hypothesis testing. These analyses were performed with Prism version 9.0.0. *Post-hoc* power calculations were performed using G power 3.1.9.4.

In addition, we conducted an analysis to evaluate risk factors for reporting changes in either period timing or flow after vaccination, including age, cycle length, the timing of vaccination relative to estimated day of ovulation and pre-existing gynecological conditions. The 4 outcome variables (Flow after dose 1; Timing after dose 1; Flow after dose 2; Timing after dose 2) were nominal (3 categories) with intrinsic order (Timing: earlier than usual/on time/later than usual; Flow: lighter than usual/same as usual/heavier than usual) thus we fit ordinal logistic regression models without proportional odds assumption using the R package “VGAM” (21).

We first conducted a series of exploratory univariable analyses on responses for which complete data were available (dose 1, *n* = 1,012; dose 2, *n* = 635), investigating associations between each of the outcome and risk factors (age, cycle length, endometriosis, heavy menstrual bleeding, uterine fibroids, PCOS, vaccine brand and the timing of vaccination in the cycle). We considered two variables for the timing of vaccination relative to the predicted day of ovulation: (i) a continuous variable with 0 corresponding to the estimated day of ovulation, and (ii) a factor variable with 3 levels (before ovulation (<-2 days); during ovulation (>-3 to <5 days) and after ovulation (>4 days). Continuous variables were scaled prior to analysis. Second, we retained all variables significant at the false discovery rate (FDR) threshold (FDR-corrected *P* < 0.05) (22) for consideration in multivariable analyses. Each multivariable model was adjusted for potential confounders, which were defined as variables significant at the FDR threshold in the univariable analyses and with a potential confounding (but not mediating) effect according to directed acyclic graphs. Estimates and confidence intervals on the log-odds scale were converted to odds ratios for reporting.

### Patient and public involvement

This study was carried out as a result of a number of unsolicited messages from members of the public, who felt that they had experienced a change to their periods following COVID-19 vaccination, and thought that more research should be done into this. The ordinal logistic regression analysis was added as an exploratory analysis following a series of webinars on COVID-19 vaccination and reproductive health issues, because the potential for people with pre-existing gynecological diagnoses to experience worse menstrual effects emerged as a common concern in the audience questions.

## Results

### COVID-19 vaccination is associated with a delay to the next period in spontaneously cycling individuals

**Figure 2:**
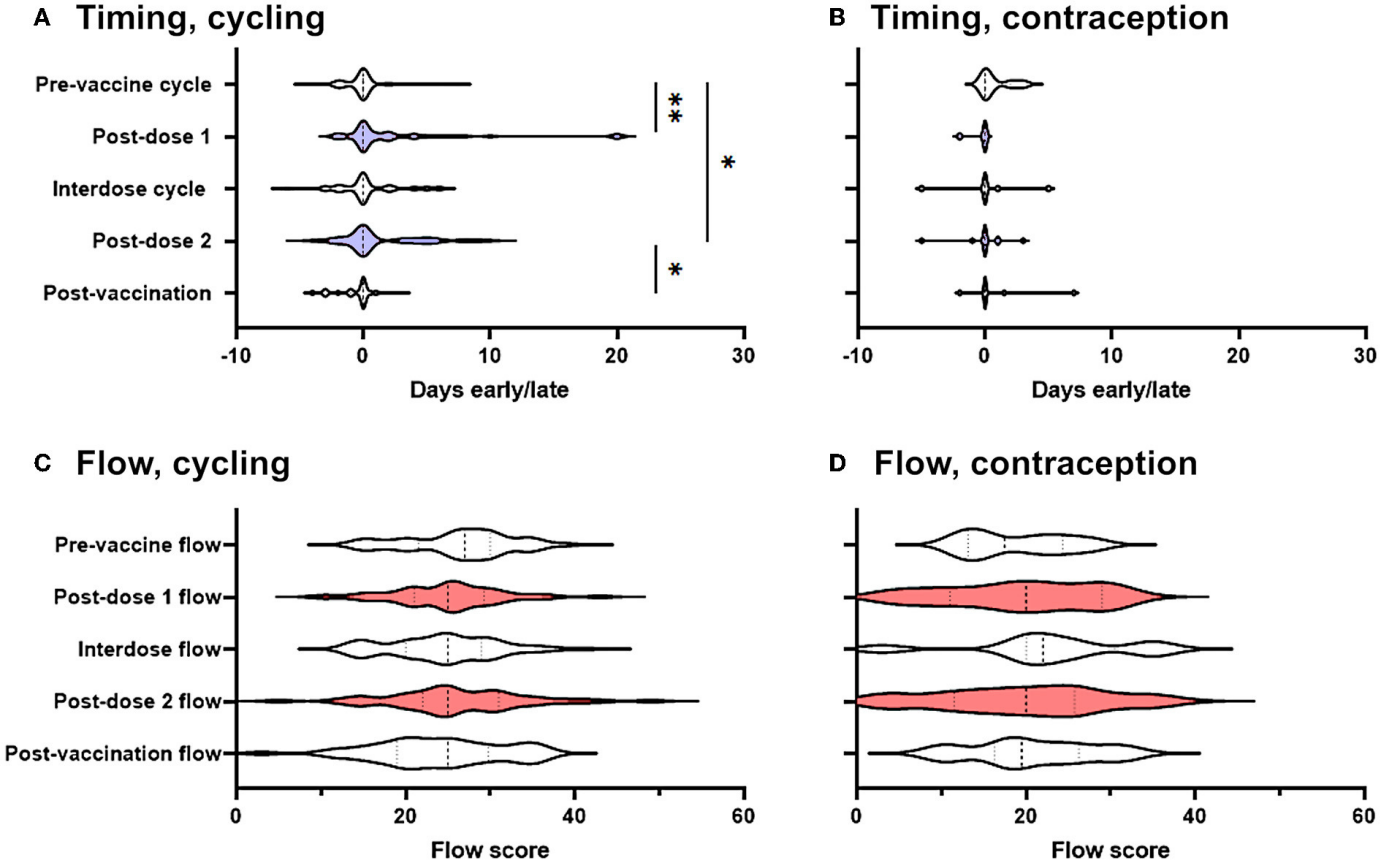
Effect of COVID-19 vaccination on menstrual timing and flow in a prospectively-recruited cohort. **(A,B)** Violin plots showing the distribution by which periods or withdrawal bleeds began in pre-vaccine cycles, the cycle following dose 1 of the COVID-19 vaccine, interdose cycles, the cycle following dose 2 of the COVID-19 vaccine, and subsequent cycles, where “0” denotes the period or withdrawal bleed beginning on the expected day, negative numbers denote days early and positive numbers days late. Data for spontaneously cycling **(A)** and participants on hormonal contraception **(B)** are shown. **(C,D)** Violin plots showing the distribution of flow scores for periods or withdrawal bleeds in pre-vaccine cycles, the cycle following dose 1 of the COVID-19 vaccine, interdose cycles, the cycle following dose 2 of the COVID-19 vaccine, and subsequent cycles. Data for spontaneously cycling **(C)** and participants on hormonal contraception **(D)** are shown. The post-vaccine cycles are shaded as a visual aid. Statistical testing with a mixed effects model, with Tukey's test for multiple comparisons. * adjusted *p* < 0.05, ** adjusted *p* < 0.01.

For each cycle, participants in the prospective cohort recorded whether their period had come on the day they had expected it, and if not how many days early or late it was: we began by using these records to determine whether periods occurred significantly earlier or later than expected following COVID-19 vaccination. Considering only those who were not using hormonal contraception (“spontaneously cycling”), we found that the period after both the first and the second dose of the vaccine came significantly later than expected, compared to pre-vaccine cycles. Compared to the day on which the participant had expected their period, pre-vaccine periods occurred on average 0.17 days early, whereas the period following dose one occurred a mean of 2.3 days late (significantly different from pre-vaccination cycles at *p*' = 0.0045) and the period following dose two occurred a mean of 1.3 days late (significantly different from pre-vaccination cycles at *p*' = 0.041) (Figure 2A). Periods following unvaccinated cycles between doses of vaccine (“interdose cycles”) and following the unvaccinated cycle after dose 2 (“post-vaccination cycles”) occurred a mean of 0.3 days late and 0.47 days early, respectively, values which were not significantly different from the pre-vaccination mean. Therefore, in spontaneously cycling individuals, vaccination was associated with a delay to the subsequent period, but periods returned to coming at the expected time in unvaccinated cycles.

Although this finding was in line with that from a large and well-designed study carried out in the USA, which also detected delays to the period following dose two of the vaccine (14), the delays we found were somewhat larger. We therefore considered the possibility that those participants who had returned their journals were more likely to have noticed a change than those who did not, inflating the magnitude and significance of post-vaccination changes. To address this possibility, we carried out two sensitivity analyses. We generated distributions consistent with no change occurring and used them to impute the data that we would have expected to see had the participants who responded that they had stopped logging their periods because they had not noticed a change returned their journals: the differences between pre-vaccination cycle timing and post-dose 1 and -dose 2 timing remained significant at *p*' = 0.0013 and *p*' = 0.011, respectively. Even assuming that none of the participants who failed to return their journals had noticed any change, significant delays to the post-dose 1 period (*p*' = 0.0035) and the post-dose 2 period (*p*' = 0.045) remained.

Considering only those participants who were taking hormonal contraception, and considering all types of hormonal contraception together due to the small numbers of these participants (*n* = 16) we were powered to detect a delay to the subsequent period of >0.37 days (α = 0.05; β = 0.08), which was smaller than the change reported by our spontaneously cycling participants. However, among participants using hormonal contraception, we found no effect of vaccination on the timing of the subsequent withdrawal bleed (Figure 2B).

### COVID-19 vaccination is not associated with any change to menstrual flow

We found no significant change to self-reported menstrual flow in the period or withdrawal bleed following vaccination, either in spontaneously cycling participants (Figure 2C), or in those using hormonal contraception (Figure 2D).

### Commonly-reported side effects are not associated with menstrual changes

One hypothesis that has been put forward to explain the existence of menstrual changes following vaccination is that immune activation may transiently interfere with the hypothalamic-pituitary-ovarian (HPO) axis (17, 18) or with immune cells in the lining of the uterus, which control the breakdown and regeneration of this tissue (19). In either of these cases, we might consider that menstrual changes are not dissimilar to other short-term side effects of vaccination and might therefore expect menstrual changes preferentially occur in those who experienced common short-term side effects.

To explore this hypothesis, we examined correlations between self-reported side effect score and either the timing or the flow score of the subsequent period: no significant correlations were found (Supplementary Table 1). Therefore, in this small cohort we were unable to find evidence that menstrual changes correlate with other common side effects of vaccination.

### Brand of vaccine is not associated with differences in timing or flow of next period

Our findings in the prospective cohort pointed to an association between COVID-19 vaccination and a delay to the subsequent period. Participants in the prospective cohort recorded parameters, such as vaccine brand and timing of vaccination within the menstrual cycle, that would allow us to begin to determine the mechanism by which these changes occurred, but the number of participants in this cohort was not sufficient to address these questions. Therefore, we also recruited a retrospective cohort, who had already been vaccinated but had a record of the dates and flow of their periods, and the date or dates of vaccination. Because this cohort is likely to be enriched for people who noticed a change to their cycle, we cannot use their reports to estimate the frequency or magnitude of post-vaccination menstrual changes. However, we can use the data to test hypotheses about how COVID-19 vaccination and menstrual changes may be connected.

**Figure 3:**
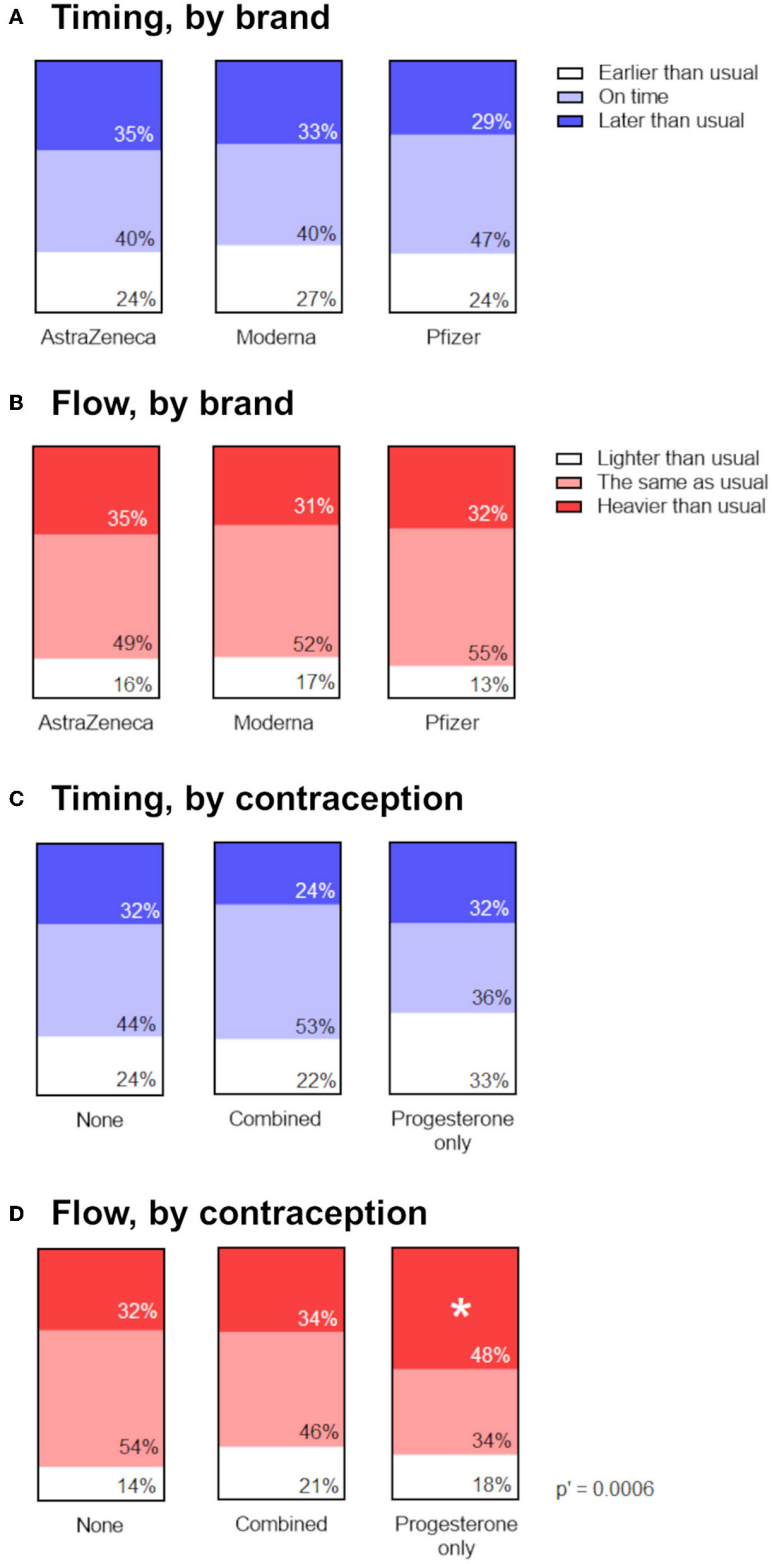
Associations between menstrual changes and brand of vaccine, or use of hormonal contraception. **(A,B)** Proportional area charts depicting the proportion of participants who reported a change to the timing **(A)** or flow **(B)** of the period following vaccination, stratified by brand of vaccine. **(C,D)** Proportional area charts depicting the proportion of participants who reported a change to the timing **(C)** or flow **(D)** of the period following vaccination, stratified by type of hormonal contraception. Statistical testing with a Chi squared test with Holm-Bonferroni sequential correction for multiple hypothesis testing. Adjusted *p*-values are shown; * denotes categories in which the standardized residual is greater than the critical value (1.96).

To determine whether menstrual changes occur as a result of a particular vaccine ingredient or approach (mRNA vs. adenovirus-vectored), we looked for associations between the proportion of respondents reporting a change in the timing (Figure 3A) or flow (Figure 3B) of the period following their vaccination, stratified by brand of vaccine received. Reports of menstrual changes to the Yellow Card surveillance scheme have not been associated with any particular brand or approach of COVID-19 vaccine (1) and in line with this, we found no association between brand of vaccine received and self-reported change to timing or flow of the next period.

### Participants using progesterone-only contraception were more likely to report heavier flow

If vaccine-associated changes to periods occur as result of transient perturbations to the HPO axis (17, 18), we might expect that people in whom exogenous ovarian hormones are supplied by hormonal contraception would be less likely to experience a menstrual change following vaccination. Our findings from the prospective cohort were in line with this, since participants using hormonal contraception did not report the change to the timing of their periods that spontaneously cycling participants did. However, because only 16 participants in this cohort were using hormonal contraception, we were unable to stratify them by combined compared to progesterone-only contraceptives. Therefore, we used the larger retrospective cohort to examine associations between the proportion of participants reporting a change in the timing or flow of the period following their vaccination, stratified by type of hormonal contraception, or none.

Examining reports of the timing of post-vaccine periods in the retrospective cohort, we did find some evidence that use of combined, but not progesterone-only, contraception was associated with being less likely to report a later-than-expected post-vaccination period (Figure 3C). The unadjusted *p*-value for the association was 0.049, but following adjustment for multiple hypothesis testing, this was no longer significant. However, we did find that participants on progesterone-only contraception were significantly more likely to report that the flow of their post-vaccination period was heavier than usual, compared to participants on combined or no hormonal contraception, even after adjustment for multiple hypothesis testing (Figure 3D).

### Timing of vaccination within the menstrual cycle does not have a clear effect on timing or flow of next period

Perturbation of the HPO axis in the follicular phase of the menstrual cycle has the potential to delay ovulation, thus delaying the subsequent period (20). Therefore, if changes to the timing of periods occur in spontaneously cycling individuals because COVID-19 vaccination impacts this, we might expect to see later than usual periods associated primarily with vaccination before ovulation.

**Figure 4:**
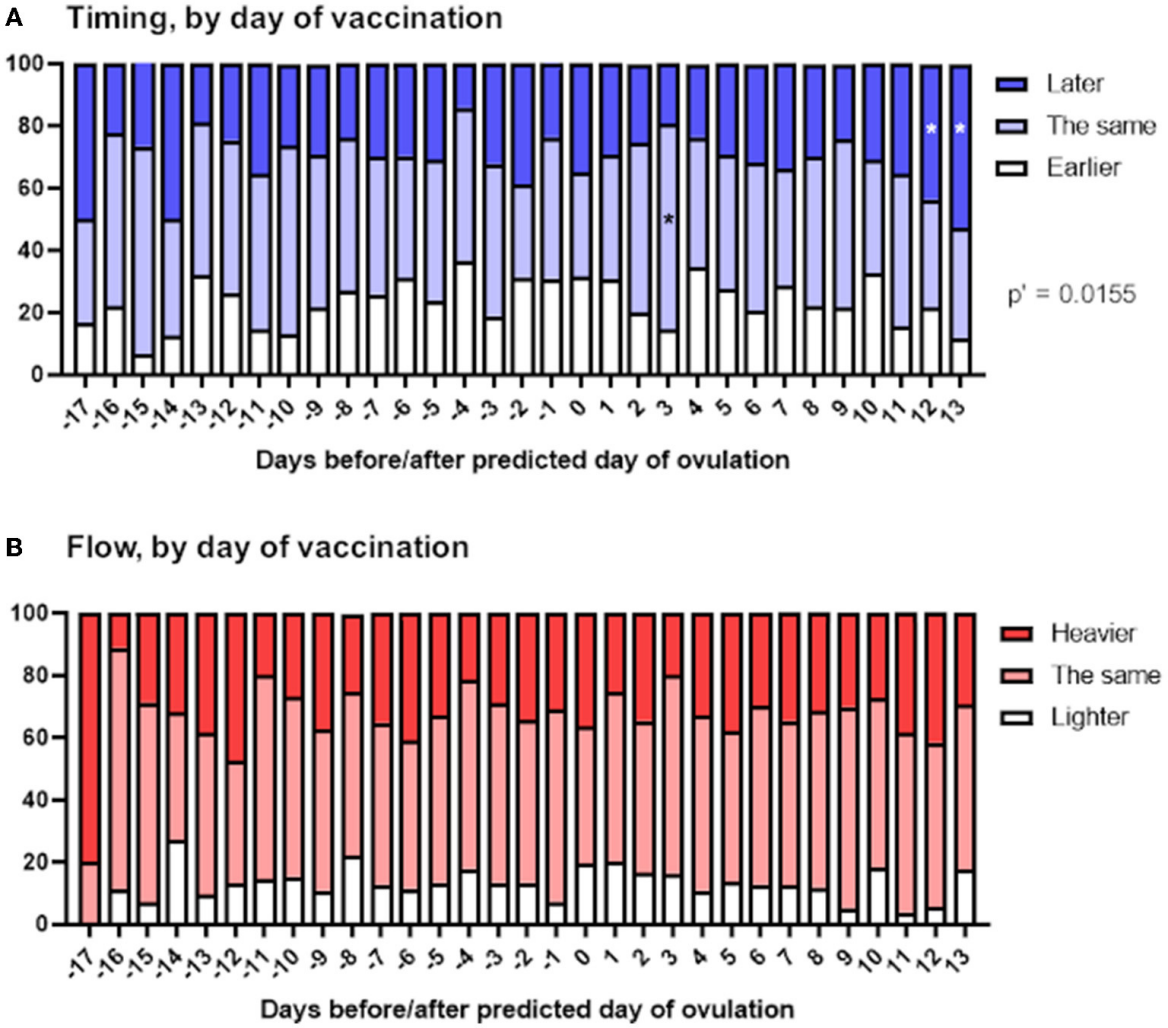
Associations between menstrual changes and timing of vaccination within the menstrual cycle. The proportional area charts depict the proportion of respondents who reported a change to the timing **(A)** or flow **(B)** of the period following vaccination, stratified by day of the menstrual cycle on which the vaccine was given, relative to the predicted day of ovulation. Statistical testing with a Chi squared test with Holm-Bonferroni sequential correction for multiple hypothesis testing. Adjusted *p*-values are shown; * denotes categories in which the standardized residual is greater than the critical value (1.96).

To examine this, we stratified reports of the timing and flow of the next period depending on the day of the menstrual cycle on which the vaccine was given, relative to predicted day of ovulation. We found a significant association between timing of vaccination within the menstrual cycle and the timing of the next period (Figure 4A). Examination of the standardized residuals revealed that this association was due to respondents vaccinated in the last 2 days of their menstrual cycle, who were more likely to report that their next period was late. This is perhaps unsurprising, since these individuals, by definition, were unlikely to report their period as arriving early, thus skewing the reports in these individuals toward later periods. We found no association between timing of vaccination and flow of the next period (Figure 4B).

### After dose 2, endometriosis is associated with an earlier period while PCOS is associated with lighter flow

A number of people have approached us with the concern that, since they already experience heavy or otherwise difficult periods because of their pre-existing conditions, any menstrual changes following COVID-19 vaccination might be more pronounced for them. Our conversations suggest that this is a major contributor to vaccine hesitancy in this group. To address this concern, we undertook ordinal logistic regression analyses to examine the effect of having a pre-existing diagnosis of abnormal menstrual bleeding, heavy menstrual bleeding, endometriosis, polycystic ovaries or uterine fibroids.

**Figure 5:**
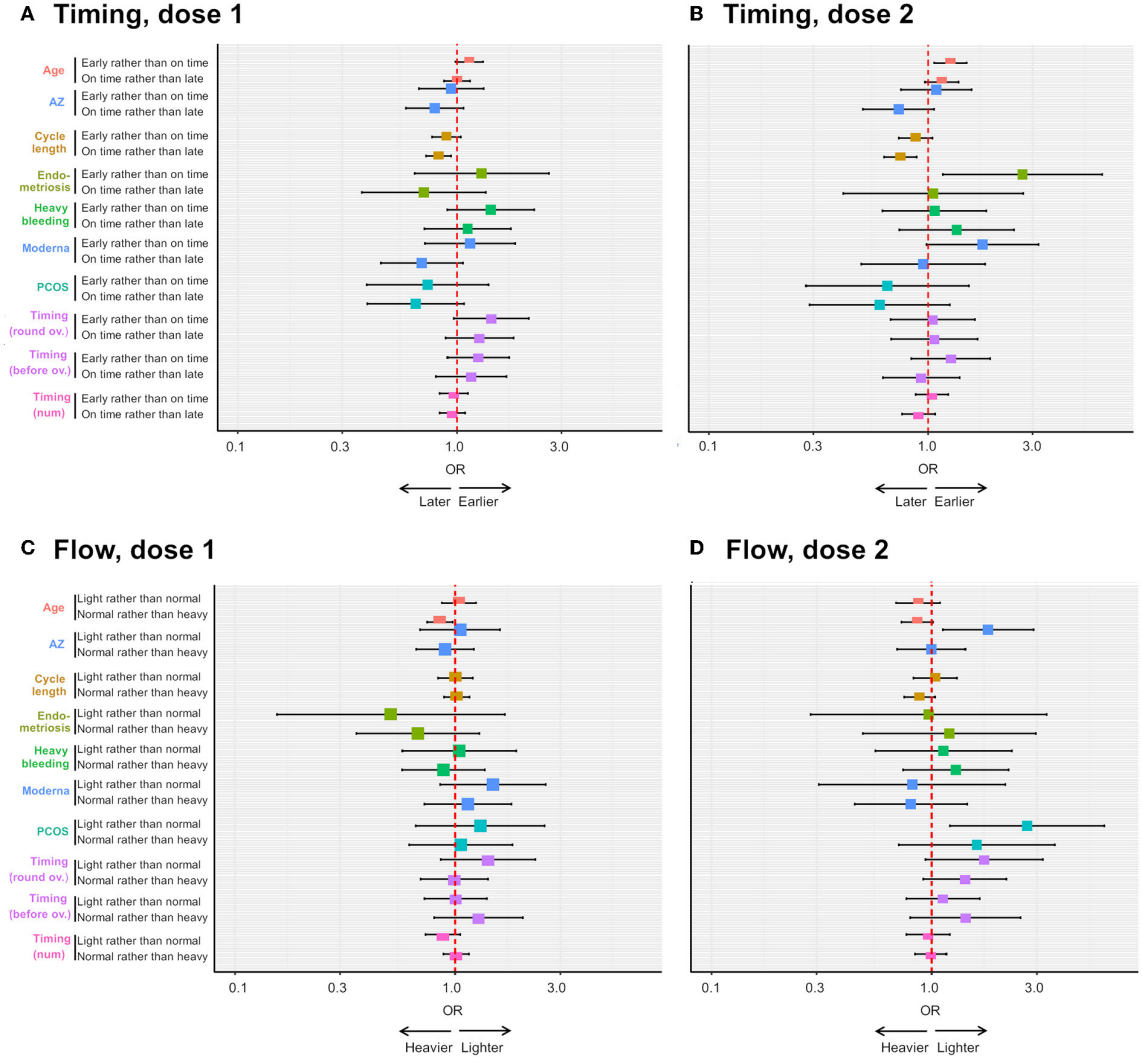
Ordinal logistic regression analyses on the effects of pre-existing gynecological condition and other factors on the risk of reporting menstrual changes. The forest plots depict the odds ratio (OR) of an earlier than usual (compared to on time) or on time (compared to later than usual) period following dose 1 **(A)** or dose 2 **(B)** of COVID-19 vaccine, depending on respondent age, receipt of the AstraZeneca (AZ) or Moderna vaccine, respondent cycle length, respondent having endometriosis, heavy menstrual bleeding (“heavy bleeding”) or PCOS, timing of vaccination as a continuous variable (“timing (num)”) or timing of vaccination between 3 days before and 4 days after ovulation (“timing (round ov.)”) or timing of vaccination more than 4 days after ovulation (“timing (after ov.)”). The OR of a lighter than usual (compared to same as usual) or a same as usual (compared to heavier than usual) period following dose 1 or dose 2 of COVID-19 vaccine is shown in **(C,D)**, respectively, for the same explanatory variables.

After dose 1 (*n* = 1,012), neither period flow nor period timing is associated with endometriosis, PCOS, uterine fibroids or heavy menstrual bleeding in this sample (Supplementary Tables 2, 3). Rather, we find that (i) older respondents are more likely to report heavier than usual flow: an increase of age of 1SD is associated with a 15% decrease in the odds of reporting normal rather than heavier than usual flow [OR = 0.85; 95 CI = (0.75; 0.97), Figure 5C] and (2) respondents with longer cycles are more likely to report later than usual periods: an increase of 1SD in cycle length is associated with a decrease of 18% in the odds of reporting periods on time compared to later than usual [OR = 0.82; 95 CI = (0.72; 0.94), Figure 5A].

After dose 2 (*n* = 635), endometriosis is associated with more than twice the odds of reporting earlier than usual, rather than on time periods [OR = 2.70; 95 CI = (1.17; 6.24), Supplementary Table 4, Figure 5B], and this effect remains in a multivariate model, after adjusting for significant associations with age and cycle length [OR = 2.6; 95 CI = (1.14; 6.11), Supplementary Table 6]. However, confidence intervals are large, as there are only a few individuals with endometriosis in this sub-sample (*n* = 23, 3.7%), suggesting the size of the effect might greatly vary in other samples. Further, PCOS and AstraZeneca are associated with an increased odds of reporting lighter than usual, rather than usual, flow [OR = 2.7; 95 CI = (1.12; 6.08) and OR = 1.8; 95 CI = (1.12; 2.90), respectively, Supplementary Table 5, Figure 5D]. We were not able to test associations with uterine fibroids as our sample for this analysis contained only 13 individuals with this condition. Finally, we found that older respondents are at an increased risk of reporting earlier than usual periods [OR = 1.23; 95 CI = (1.03; 1.47)], and those with longer cycles are less likely to report periods on time rather than later than usual [OR = 0.76; 95 CI = (0.637; 0.637), Supplementary Table 4, Figure 5B].

## Discussion

Here we report that, in a prospectively-recruited cohort of 79 participants, periods occurred later than expected after both the first and second dose of the COVID-19 vaccine among spontaneously cycling participants. This finding was robust to a sensitivity analysis to account for the likelihood that participants who noticed a change may be more motivated to return their journals. Importantly, periods returned to coming at the expected time in the interdose and post-vaccine cycles in this cohort. These findings are in line with those from a recent study in the USA, which reported increases in cycle length associated with receiving either the second dose of vaccine, or both doses in the same cycle, but again noted that cycles rapidly returned to normal (14). Interestingly, we did not find a change to the perceived timing of post-vaccination periods among participants using hormonal contraception, even though we were powered to detect such changes.

We were unable to identify any change in self-reported flow associated with COVID-19 vaccination, and this is in contrast to findings from a Norwegian study, which has appeared in preprint (23). This study used mobile-phone questionnaires to retrospectively solicit reports of menstrual changes from 5,688 women aged 18–30 years who had been recruited prospectively into the Norwegian Young Adult Cohort. High levels of variation were reported even in unvaccinated cycles, with 37.8% of participants reporting at least one difference from their personal norm in their pre-vaccination cycles. However, the study was still able to identify reports of heavier than normal bleeding more commonly associated with vaccination. Our prospectively-recruited cohort was smaller than the Norwegian cohort and this may have constrained our ability to detect real changes in menstrual flow. On the other hand, participants in our cohort recorded data in real time, whereas those in the Norwegian cohort were asked to recall their experiences, so it is also possible that the difference reported in the Norwegian cohort is at least partially influenced by recall bias.

Because the participants in our prospective cohort were recruited before vaccination and asked to record their experiences in real time, the findings from this cohort are likely to be minimally affected by recruitment and recall bias. The repeated measures design aims to avoid bias introduced by systematic differences that are likely to exist between individuals who choose to receive the vaccine and those who do not, although a limitation of this approach is that there is no unvaccinated comparison cohort. Some bias may have been introduced because it is likely that the participants who did not return their sheets did so not at random, although we did attempt to account for this with two sensitivity analyses. Some bias may also have been introduced because “expected date of period” and “menstrual flow” are at least partially subjective, so there is the potential for participants' expectations to impact their reports.

Although the prospective arm of the study allows us to make conclusions about a causal link between vaccination and changes to the menstrual cycle, a major weakness of this arm is that the cohort is small, and therefore is not powered to explore hypotheses about the mechanism controlling menstrual changes following vaccination. To compensate for this, we recruited a second, larger cohort retrospectively. In the first analysis of this cohort of 1,273 participants, we were unable to detect an association between brand of vaccine received and changes to timing or flow of the next period. The observation that there is no difference in reports following vaccination with mRNA compared to adenovirus-vectored vaccines suggests that any effects are mediated by activation of the immune response, rather than by any particular vaccine approach. This is in line with reports that menstrual changes can occur in association with other immune challenges, including HPV vaccination (13) and SARS-CoV-2 infection (15, 16).

It has been suggested that cytokine production as part of the immune response, whether from vaccination (24, 25) or infection (26), may transiently interfere with the HPO axis and thus the production of ovarian hormones that drive the menstrual cycle (17, 18). To explore the possibility that vaccine-associated menstrual delays observed in the prospective cohort were mediated in this way, we examined the effect of hormonal contraception and timing of vaccination within the cycle on participants' reports: if vaccine effects on menstrual timing are mediated by ovarian hormones, we might expect that supplying these exogenously, as in hormonal contraception, would protect against experiencing changes in cycle length. We did detect a signal suggesting that participants taking combined hormonal contraception may be less likely to experience a post-vaccination delay to their next bleed, compared to those on progesterone-only or no contraception, although this was not significant following adjustment for multiple hypothesis testing. This finding is in line with the results of a survey of menstrual experiences following COVID-19 vaccination, which also found that respondents using combined, but not progesterone-only, contraceptives were less likely to report post-vaccination menstrual changes (27). We also found that participants using progesterone-only contraception were significantly more likely to report a heavier than usual withdrawal bleed following vaccination, and this is in line with the results of another survey, which also found people using hormonal contraception were more likely to report heavier than usual flow post-vaccination, although this study did not distinguish between combined and progesterone-only contraception (28).

The indications from this study and others (27) that combined hormonal contraception protects against vaccination-associated menstrual changes, but progesterone-only contraception does not, provides some support for the idea that ovarian hormones, and thus the HPO axis, may mediate the effects of COVID-19 vaccination on the menstrual cycle.

To further test this idea, we also examined the effect of vaccine timing within the menstrual cycle on the subsequent period: because perturbation of the HPO axis in the follicular phase has the potential to delay ovulation, and thus the subsequent period, we would expect to see delays associated mainly with vaccination at this time. We were unable to find such an association, however it is worth noting that the day of ovulation was predicted using a crude method based on normal cycle length: future studies in which day of ovulation is defined using basal body temperature or urine LH measurement will be able to determine date of vaccination relative to ovulation with greater accuracy and thus may be able to detect associations we were unable to here.

In response to patient and public interest, we undertook further ordinal logistic regression analyses to examine the effect of having a pre-existing diagnosis of a gynecological condition on the likelihood that participants would report changes to menstrual timing or flow. We found that, after the second dose, respondents with a pre-existing diagnosis of endometriosis were more likely to report an earlier than usual period. Since this finding was based on only a small number of individuals (23 who had received both doses) the confidence intervals were wide, and so the finding should be replicated before any causal association is suggested. In general, endometriosis is associated with irregular periods, and particularly shorter cycles (29) and one possibility is that respondents might be more likely to notice and report a symptom that they are more likely to experience even in the absence of vaccination. After the second dose, we also found individuals with PCOS were more likely to report a lighter than usual period. Since PCOS can be associated with light periods (30), again it is possible that respondents are more likely to report a change which they commonly experience. It has also been reported that androgen excess, which is a feature of PCOS (30), may be a risk for more severe SARS-CoV-2 disease (31, 32) so it could potentially be the case that PCOS patients are also more at risk from side effects of COVID-19 vaccines.

Interestingly this analysis also found that people who had received AstraZeneca were more likely to report a lighter than usual period, but only following their second dose. This effect was not due recipients of AstraZeneca being older on average, since age was controlled for in the analysis. At the time of survey, it was widely known that AstraZeneca was associated with a rare clotting side effects (33): this may have affected respondents' perceptions of their periods, although in this case we might have expected to see reports of heavier than usual, rather than lighter than usual periods, and not specifically associated with the second dose. The finding that participants reported this change only in association with the second dose suggests it may represent an immune-mediated effect, similar to other commonly-reported non-menstrual side effects that are also more prominent following the second dose, and the finding that this was associated only with AstraZeneca could be in line with reports that side effects are more commonly associated with the AstraZeneca than the Pfizer vaccine (34).

In summary, we report here that, in a prospectively-recruited cohort, COVID-19 vaccination is associated with a delay to the subsequent menstrual period in spontaneously cycling individuals, but the timing of periods returns to normal in unvaccinated cycles. We were unable to detect a change in flow associated with COVID-19 vaccination. Our finding that individuals on hormonal contraception did not experience a change to the timing of post-vaccination periods, supported by findings from the retrospective cohort and other studies (27) that combined hormonal contraception seems to protect against reporting menstrual changes, suggests that post-vaccination menstrual changes may occur as a result of temporary perturbation of the HPO axis, but further research is needed to confirm this. Our finding that individuals with endometriosis or PCOS may be more likely to notice a change to their periods warrants further investigation: in the interim, we emphasize that these findings should not be used to counsel people who have these diagnoses against vaccination. Indeed, it is important for those who are particularly concerned about changes to their menstrual cycles to be reminded that SARS-CoV-2 infection itself may cause this (15, 16).

## Data availability statement

The datasets presented in this study can be found in online repositories. The names of the repository/repositories and accession number(s) can be found below: data collection tools and cleaned, fully anonymized data, together with Prism analysis files are available from the Open Science Framework at https://osf.io/upbyg/ (prospective cohort) and https://osf.io/6jf4u/ (retrospective cohort). Code is available from https://osf.io/jtyau/files/ and https://github.com/ataquette/Covid-vaccination_Male#covid-vaccination_male.

## Ethics statement

The studies involving human participants were reviewed and approved by the Research Governance and Integrity Team at Imperial College London, study number 21IC6988. The patients/participants provided their written informed consent to participate in this study.

## Author contributions

VM designed the study. VM and EW recruited participants and collected data. VM and AA analyzed data and wrote the manuscript. All authors had the opportunity to read and edit the manuscript and have approved it for submission.

## Funding

VM's salary was paid by the Wellcome Trust (WT105677; to August 2021) and the preterm birth charity Borne (from August 2021).

## Conflict of interest

The authors declare that the research was conducted in the absence of any commercial or financial relationships that could be construed as a potential conflict of interest.

## Publisher's note

All claims expressed in this article are solely those of the authors and do not necessarily represent those of their affiliated organizations, or those of the publisher, the editors and the reviewers. Any product that may be evaluated in this article, or claim that may be made by its manufacturer, is not guaranteed or endorsed by the publisher.
