# Supplementary material for: Effect of COVID-19 vaccination on the timing and flow of menstrual periods in two cohorts
Source: Front Reprod Health. 2022 Jul 25;4:952976. doi: 10.3389/frph.2022.952976 (PMC9580734; doi:10.3389/frph.2022.952976)
Supplement: Supplementary file 1 [file Data_Sheet_1.PDF]

Supplementary Material

Side effect score *vs*	Dose	Contraception	Spearman's r	p	p'
Timing	1	No	-0.05880	0.3575	1.00
Flow	1	No	0.1014	0.2588	1.00
Timing	2	No	0.01375	0.4607	1.00
Flow	2	No	0.07823	0.2870	1.00
Timing	1	Yes	-0.4648	0.3333	1.00
Flow	1	Yes	-0.4926	0.1286	1.00
Timing	2	Yes	0.2309	0.2268	1.00
Flow	2	Yes	0.3159	0.1338	1.00

Supplementary Table 1. Correlations between side effect score and timing or flow of the subsequent period, in prospective cohort participants not using or using hormonal contraception.

Supplementary Tables 2 – 5 are attached below and show univariable analysis of period timing after dose 1 (Supplementary Table 2), flow after dose 1 (Supplementary Table 3), timing after dose 2 (Supplementary Table 4) and flow after dose 2 (Supplementary Table 5).

Supplementary Table 6 is attached below and shows multivariable analysis of period timing after dose 2.

Supplementary Table 2

Timing dose 1 | Univariable models adjusted p-values

term	OR	CILow	CIHigh	statistic	p.value	q.value
(Intercept):earlier rather than on time	0.286	0.246	0.332	-16.500	0.000	0.000
(Intercept):on time rather than later	2.420	2.113	2.772	12.771	0.000	0.000
age_scaled:earlier rather than on time	1.139	0.983	1.320	1.736	0.083	0.157
age_scaled:on time rather than later	1.002	0.875	1.148	0.035	0.972	0.972
(Intercept):earlier rather than on time	0.285	0.246	0.331	-16.556	0.000	0.000
(Intercept):on time rather than later	2.436	2.126	2.792	12.801	0.000	0.000
cycle_length_scaled:earlier rather than on time	0.896	0.770	1.044	-1.410	0.158	0.244
cycle_length_scaled:on time rather than later	0.824	0.721	0.942	-2.837	0.005	0.010
(Intercept):earlier rather than on time	0.283	0.243	0.329	-16.319	0.000	0.000
(Intercept):on time rather than later	2.456	2.137	2.821	12.694	0.000	0.000
endometriosis:earlier rather than on time	1.297	0.639	2.631	0.721	0.471	0.585
endometriosis:on time rather than later	0.706	0.368	1.353	-1.049	0.294	0.405
(Intercept):earlier rather than on time	0.275	0.235	0.322	-16.012	0.000	0.000
(Intercept):on time rather than later	2.392	2.074	2.758	11.988	0.000	0.000
heavy_menstrual_bleeding:earlier rather than on time	1.426	0.902	2.253	1.519	0.129	0.211
heavy_menstrual_bleeding:on time rather than later	1.120	0.709	1.768	0.486	0.627	0.697
(Intercept):earlier rather than on time	0.288	0.248	0.334	-16.346	0.000	0.000
(Intercept):on time rather than later	2.412	2.104	2.766	12.631	0.000	0.000
uterine_fibroids:earlier rather than on time	0.651	0.188	2.254	-0.677	0.498	0.586
uterine_fibroids:on time rather than later	1.161	0.414	3.252	0.283	0.777	0.797
(Intercept):earlier rather than on time	0.291	0.250	0.339	-15.837	0.000	0.000
(Intercept):on time rather than later	2.496	2.168	2.875	12.702	0.000	0.000
pcos:earlier rather than on time	0.735	0.387	1.397	-0.938	0.348	0.464
pcos:on time rather than later	0.647	0.389	1.077	-1.676	0.094	0.164
(Intercept):earlier rather than on time	0.286	0.237	0.346	-12.860	0.000	0.000
(Intercept):on time rather than later	2.703	2.261	3.232	10.913	0.000	0.000
brand_dose1_AstraZeneca:earlier rather than on time	0.941	0.671	1.322	-0.348	0.728	0.766
brand_dose1_AstraZeneca:on time rather than later	0.792	0.584	1.073	-1.506	0.132	0.211
brand_dose1_Moderna:earlier rather than on time	1.150	0.715	1.849	0.577	0.564	0.645
brand_dose1_Moderna:on time rather than later	0.691	0.449	1.065	-1.673	0.094	0.164
(Intercept):earlier rather than on time	0.286	0.246	0.331	-16.563	0.000	0.000
(Intercept):on time rather than later	2.420	2.113	2.771	12.784	0.000	0.000
vaccine_timing_dose1_scaled:earlier rather than on time	0.966	0.833	1.120	-0.456	0.648	0.701
vaccine_timing_dose1_scaled:on time rather than later	0.953	0.832	1.091	-0.702	0.483	0.585
(Intercept):earlier rather than on time	0.240	0.179	0.322	-9.499	0.000	0.000
(Intercept):on time rather than later	2.054	1.603	2.631	5.692	0.000	0.000
vaccine_timing_dose1_Before ovulation:earlier rather than on time	1.160	0.800	1.683	0.782	0.434	0.560
vaccine_timing_dose1_Before ovulation:on time rather than later	1.253	0.906	1.733	1.364	0.172	0.256
vaccine_timing_dose1_During ovulation :earlier rather than on time	1.439	0.970	2.135	1.809	0.070	0.141
vaccine_timing_dose1_During ovulation :on time rather than later	1.268	0.888	1.812	1.307	0.191	0.273

Supplementary Table 3

Flow after dose 1 | Univariable models adjusted p-values

term	OR	CILow	CIHigh	statistic	p.value	q.value
**Age**						
(Intercept):lighter rather than same	0.152	0.126	0.182	-20.252	0.000	0.000
(Intercept):normal rather than heavy	2.265	1.981	2.591	11.930	0.000	0.000
age_scaled:lighter rather than same	1.041	0.868	1.248	0.433	0.665	0.831
age_scaled:normal rather than heavy	0.852	0.746	0.974	-2.347	0.019	0.040
**Cycle length**						
(Intercept):lighter rather than same	0.151	0.126	0.182	-20.297	0.000	0.000
(Intercept):normal rather than heavy	2.265	1.981	2.588	11.986	0.000	0.000
cycle_length_scaled:lighter rather than same	1.004	0.837	1.205	0.045	0.964	0.971
cycle_length_scaled:normal rather than heavy	1.017	0.889	1.162	0.240	0.810	0.938
**Endometriosis**						
(Intercept):lighter rather than same	0.155	0.129	0.186	-19.812	0.000	0.000
(Intercept):normal rather than heavy	2.303	2.008	2.641	11.942	0.000	0.000
endometriosis:lighter rather than same	0.511	0.155	1.679	-1.107	0.268	0.447
endometriosis:normal rather than heavy	0.679	0.357	1.290	-1.183	0.237	0.412
**Heavy menstrual bleeding**						
(Intercept):lighter rather than same	0.151	0.124	0.183	-19.250	0.000	0.000
(Intercept):normal rather than heavy	2.293	1.991	2.642	11.508	0.000	0.000
heavy_menstrual_bleeding:lighter rather than same	1.044	0.576	1.894	0.143	0.887	0.958
heavy_menstrual_bleeding:normal rather than heavy	0.885	0.573	1.366	-0.552	0.581	0.750
**Fibroids**						
(Intercept):lighter rather than same	0.153	0.128	0.184	-20.062	0.000	0.000
(Intercept):normal rather than heavy	2.277	1.989	2.607	11.941	0.000	0.000
uterine_fibroids:lighter rather than same	0.362	0.048	2.736	-0.984	0.325	0.500
uterine_fibroids:normal rather than heavy	0.753	0.294	1.931	-0.591	0.555	0.750
**PCOS**						
(Intercept):lighter rather than same	0.148	0.123	0.180	-19.663	0.000	0.000
(Intercept):normal rather than heavy	2.255	1.964	2.590	11.527	0.000	0.000
pcos:lighter rather than same	1.300	0.663	2.548	0.765	0.444	0.658
pcos:normal rather than heavy	1.064	0.620	1.826	0.226	0.821	0.938
**Vaccine Brand**						
(Intercept):lighter rather than same	0.142	0.112	0.181	-15.920	0.000	0.000
(Intercept):normal rather than heavy	2.303	1.938	2.736	9.473	0.000	0.000
brand_dose1_AstraZeneca:lighter rather than same	1.053	0.694	1.598	0.243	0.808	0.938
brand_dose1_AstraZeneca:normal rather than heavy	0.901	0.667	1.215	-0.685	0.493	0.704
brand_dose1_Moderna:lighter rather than same	1.486	0.857	2.576	1.412	0.158	0.315
brand_dose1_Moderna:normal rather than heavy	1.144	0.726	1.801	0.579	0.563	0.750
**Timing of vaccine (continuous time)**						
(Intercept):lighter rather than same	0.150	0.125	0.181	-20.254	0.000	0.000
(Intercept):normal rather than heavy	2.265	1.981	2.588	11.986	0.000	0.000
vaccine_timing_dose1_scaled:lighter rather than same	0.880	0.734	1.056	-1.375	0.169	0.315
vaccine_timing_dose1_scaled:normal rather than heavy	1.010	0.884	1.154	0.145	0.885	0.958
**Timing of vaccine (discrete time)**						
(Intercept):lighter rather than same	0.123	0.084	0.178	-11.033	0.000	0.000
(Intercept):normal rather than heavy	2.264	1.759	2.914	6.349	0.000	0.000
vaccine_timing_dose1_Before ovulation:lighter rather than same	1.277	0.804	2.029	1.036	0.300	0.480
vaccine_timing_dose1_Before ovulation:normal rather than heavy	1.006	0.727	1.392	0.037	0.971	0.971
vaccine_timing_dose1_During ovulation :lighter rather than same	1.409	0.860	2.309	1.362	0.173	0.315
vaccine_timing_dose1_During ovulation :normal rather than heavy	0.991	0.696	1.412	-0.049	0.961	0.971

Supplementary Table 4

Timing dose 2 | Univariable models adjusted p-values

term	OR	CILow	CIHigh	statistic	p.value	q.value
(Intercept):earlier rather than on time	0.412	0.346	0.489	-10.072	0.000	0.000
(Intercept):on time rather than later	2.693	2.259	3.211	11.042	0.000	0.000
age_scaled:earlier rather than on time	1.267	1.066	1.506	2.689	0.007	0.014
age_scaled:on time rather than later	1.155	0.968	1.378	1.596	0.110	0.181
(Intercept):earlier rather than on time	0.419	0.353	0.497	-9.975	0.000	0.000
(Intercept):on time rather than later	2.740	2.294	3.273	11.117	0.000	0.000
cycle_length_scaled:earlier rather than on time	0.878	0.736	1.046	-1.458	0.145	0.227
cycle_length_scaled:on time rather than later	0.749	0.630	0.889	-3.294	0.001	0.002
(Intercept):earlier rather than on time	0.404	0.339	0.481	-10.158	0.000	0.000
(Intercept):on time rather than later	2.687	2.248	3.211	10.870	0.000	0.000
endometriosis:earlier rather than on time	2.702	1.171	6.239	2.329	0.020	0.038
endometriosis:on time rather than later	1.055	0.409	2.720	0.110	0.913	0.913
(Intercept):earlier rather than on time	0.418	0.349	0.500	-9.481	0.000	0.000
(Intercept):on time rather than later	2.611	2.173	3.139	10.228	0.000	0.000
heavy_menstrual_bleeding:earlier rather than on time	1.070	0.620	1.846	0.244	0.807	0.877
heavy_menstrual_bleeding:on time rather than later	1.353	0.741	2.470	0.984	0.325	0.423
(Intercept):earlier rather than on time	0.429	0.360	0.511	-9.527	0.000	0.000
(Intercept):on time rather than later	2.769	2.311	3.317	11.041	0.000	0.000
pcos:earlier rather than on time	0.653	0.277	1.536	-0.977	0.329	0.423
pcos:on time rather than later	0.602	0.288	1.259	-1.348	0.178	0.267
(Intercept):earlier rather than on time	0.387	0.308	0.486	-8.175	0.000	0.000
(Intercept):on time rather than later	3.011	2.378	3.812	9.160	0.000	0.000
brand_dose2_AstraZeneca:earlier rather than on time	1.092	0.753	1.584	0.463	0.643	0.773
brand_dose2_AstraZeneca:on time rather than later	0.735	0.505	1.068	-1.614	0.107	0.181
brand_dose2_Moderna:earlier rather than on time	1.775	0.986	3.198	1.912	0.056	0.101
brand_dose2_Moderna:on time rather than later	0.949	0.494	1.823	-0.157	0.875	0.900
(Intercept):earlier rather than on time	0.420	0.355	0.499	-9.964	0.000	0.000
(Intercept):on time rather than later	2.698	2.264	3.215	11.093	0.000	0.000
vaccine_timing_dose2_scaled:earlier rather than on time	1.041	0.878	1.235	0.462	0.644	0.773
vaccine_timing_dose2_scaled:on time rather than later	0.905	0.759	1.079	-1.112	0.266	0.369
(Intercept):earlier rather than on time	0.428	0.315	0.580	-5.463	0.000	0.000
(Intercept):on time rather than later	2.397	1.764	3.256	5.591	0.000	0.000
vaccine_timing_dose2_Before ovulation:earlier rather than on time	0.931	0.621	1.395	-0.348	0.728	0.845
vaccine_timing_dose2_Before ovulation:on time rather than later	1.271	0.840	1.922	1.135	0.256	0.369
vaccine_timing_dose2_During ovulation :earlier rather than on time	1.051	0.673	1.639	0.217	0.828	0.877
vaccine_timing_dose2_During ovulation :on time rather than later	1.069	0.680	1.682	0.290	0.772	0.869

Supplementary Table 5

Flow after dose 2 | Univariable models adjusted p-values

term	OR	CILow	CIHigh	statistic	p.value	q.value
**Age**						
(Intercept):lighter rather than same	0.154	0.122	0.194	-15.947	0.000	0.000
(Intercept):normal rather than heavy	1.937	1.643	2.285	7.860	0.000	0.000
age_scaled:lighter rather than same	0.869	0.690	1.095	-1.190	0.234	0.351
age_scaled:normal rather than heavy	0.859	0.729	1.013	-1.801	0.072	0.136
**Cycle length**						
(Intercept):lighter rather than same	0.154	0.123	0.194	-16.019	0.000	0.000
(Intercept):normal rather than heavy	1.917	1.627	2.259	7.767	0.000	0.000
cycle_length_scaled:lighter rather than same	1.038	0.828	1.301	0.324	0.746	0.817
cycle_length_scaled:normal rather than heavy	0.881	0.749	1.037	-1.524	0.127	0.209
**Endometriosis**						
(Intercept):lighter rather than same	0.155	0.123	0.195	-15.726	0.000	0.000
(Intercept):normal rather than heavy	1.900	1.609	2.245	7.550	0.000	0.000
endometriosis:lighter rather than same	0.970	0.282	3.336	-0.049	0.961	0.983
endometriosis:normal rather than heavy	1.203	0.487	2.969	0.400	0.689	0.817
**Heavy menstrual bleeding**						
(Intercept):lighter rather than same	0.152	0.120	0.194	-15.174	0.000	0.000
(Intercept):normal rather than heavy	1.864	1.568	2.215	7.067	0.000	0.000
heavy_menstrual_bleeding:lighter rather than same	1.131	0.554	2.309	0.338	0.735	0.817
heavy_menstrual_bleeding:normal rather than heavy	1.288	0.743	2.231	0.902	0.367	0.508
**PCOS**						
(Intercept):lighter rather than same	0.144	0.113	0.183	-15.782	0.000	0.000
(Intercept):normal rather than heavy	1.871	1.583	2.213	7.332	0.000	0.000
pcos:lighter rather than same	2.713	1.210	6.083	2.424	0.015	0.031
pcos:normal rather than heavy	1.603	0.708	3.631	1.131	0.258	0.371
**Vaccine Brand**						
(Intercept):lighter rather than same	0.125	0.090	0.173	-12.553	0.000	0.000
(Intercept):normal rather than heavy	1.952	1.573	2.422	6.081	0.000	0.000
brand_dose2_AstraZeneca:lighter rather than same	1.803	1.121	2.902	2.431	0.015	0.031
brand_dose2_AstraZeneca:normal rather than heavy	0.996	0.697	1.423	-0.021	0.983	0.983
brand_dose1_Moderna:lighter rather than same	0.816	0.308	2.166	-0.408	0.684	0.817
brand_dose1_Moderna:normal rather than heavy	0.805	0.447	1.449	-0.723	0.470	0.626
**Timing of vaccine (continuous time)**						
(Intercept):lighter rather than same	0.154	0.123	0.194	-16.019	0.000	0.000
(Intercept):normal rather than heavy	1.913	1.624	2.253	7.760	0.000	0.000
vaccine_timing_dose2_scaled:lighter rather than same	0.963	0.767	1.210	-0.320	0.749	0.817
vaccine_timing_dose2_scaled:normal rather than heavy	0.990	0.840	1.166	-0.120	0.905	0.958
**Timing of vaccine (discrete time)**						
(Intercept):lighter rather than same	0.113	0.071	0.179	-9.245	0.000	0.000
(Intercept):normal rather than heavy	1.662	1.246	2.218	3.454	0.001	0.001
vaccine_timing_dose1_Before ovulation:lighter rather than same	1.424	0.799	2.538	1.198	0.231	0.351
vaccine_timing_dose1_Before ovulation:normal rather than heavy	1.126	0.768	1.651	0.606	0.545	0.700
vaccine_timing_dose1_During ovulation :lighter rather than same	1.733	0.937	3.204	1.753	0.080	0.143
vaccine_timing_dose1_During ovulation :normal rather than heavy	1.416	0.915	2.190	1.561	0.119	0.203

Supplementary Table 6

Timing after dose 2 | Multivariable model

	OR	CILow	CIHigh	z	Pr(>|z|)
**(Intercept):earlier rather than on time**	**0.395**	**0.330**	**0.472**	**-10.257**	**0.000**
**(Intercept):on time rather than later**	**2.729**	**2.274**	**3.275**	**10.852**	**0.000**
**age_scaled:earlier rather than on time**	**1.230**	**1.029**	**1.470**	**2.277**	**0.023**
age_scaled:on time rather than later	1.090	0.906	1.310	0.924	0.356
cycle_length:earlier rather than on time	0.910	0.759	1.092	-1.006	0.315
**cycle_length:on time rather than later**	**0.760**	**0.637**	**0.906**	**-3.048**	**0.002**
**endometriosis:earlier rather than on time**	**2.635**	**1.137**	**6.109**	**2.260**	**0.024**
endometriosis:on time rather than later	1.039	0.402	2.682	0.079	0.937
